# Comprehensive Immunoprofiling of High-Risk Oral Proliferative and Localized Leukoplakia

**DOI:** 10.1158/2767-9764.CRC-21-0060

**Published:** 2021-10-13

**Authors:** Glenn J. Hanna, Alessandro Villa, Nikhil Mistry, Yonghui Jia, Charles T. Quinn, Madison M. Turner, Kristen D. Felt, Kathleen Pfaff, Robert I. Haddad, Ravindra Uppaluri, Scott J. Rodig, Sook-Bin Woo, Ann Marie Egloff, F. Stephen Hodi

**Affiliations:** 1 Department of Medical Oncology, Dana-Farber Cancer Institute, Boston, Massachusetts.; 2 Oral Medicine Clinic, University of California San Francisco School of Dentistry, San Francisco, California.; 3 Division of Oral Medicine and Dentistry, Harvard School of Dental Medicine, Boston, Massachusetts.; 4 Department of Pathology, Brigham & Women's Hospital, Boston, Massachusetts.; 5 Center for Immuno-Oncology, Dana-Farber Cancer Institute, Boston, Massachusetts.; 6 ImmunoProfile, Brigham & Women's Hospital and Dana-Farber Cancer Institute, Boston, Massachusetts.; 7 Division of Otolaryngology-Head and Neck Surgery, Department of Surgery, Brigham & Women's Hospital, Boston, Massachusetts.

## Abstract

Oral leukoplakia is common and may, in some cases, progress to carcinoma. Proliferative leukoplakia is a progressive, often multifocal subtype with a high rate of malignant transformation compared with the more common localized leukoplakia. We hypothesized that the immune microenvironment and gene expression patterns would be distinct for proliferative leukoplakia compared with localized leukoplakia. We summarize key clinicopathologic features among proliferative leukoplakia and localized leukoplakia and compare cancer-free survival (CFS) between subgroups. We analyze immunologic gene expression profiling in proliferative leukoplakia and localized leukoplakia tissue samples (NanoString PanCancer Immune Oncology Profiling). We integrate immune cell activation and spatial distribution patterns in tissue samples using multiplexed immunofluorescence and digital image capture to further define proliferative leukoplakia and localized leukoplakia. Among *N* = 58 patients (proliferative leukoplakia, *n* = 29; localized leukoplakia, *n* = 29), only the clinical diagnosis of proliferative leukoplakia was associated with significantly decreased CFS (HR, 11.25; *P* < 0.01; 5-year CFS 46.8% and 83.6% among patients with proliferative leukoplakia and localized leukoplakia, respectively). CD8^+^ T cells and T regulatory (Treg) were more abundant among proliferative leukoplakia samples (*P* < 0.01) regardless of degree of epithelial dysplasia, and often colocalized to the dysplasia–stromal interface. Gene set analysis identified granzyme M as the most differentially expressed gene favoring the proliferative leukoplakia subgroup (log_2_ fold change, 1.93; *P*_adj_ < 0.001). Programmed death ligand 1 (PD-L1) was comparatively overexpressed among proliferative leukoplakia samples, with higher (>5) PD-L1 scores predicting worse CFS (*P*_adj_ < 0.01). Proliferative leukoplakia predicts a high rate of malignant transformation within 5 years of diagnosis. A prominent CD8^+^ T-cell and Treg signature along with relative PD-L1 overexpression compared with localized leukoplakia provides strong rationale for PD-1/PD-L1 axis blockade using preventative immunotherapy.

**Significance:**

This is the first in-depth profiling effort to immunologically characterize high-risk proliferative leukoplakia as compared with the more common localized leukoplakia. We observed a notable cytotoxic T-cell and Treg signature with relative overexpression of PD-L1 in high-risk proliferative leukoplakia providing a strong preclinical rationale for investigating PD-1/PD-L1 axis blockade in this disease as preventative immunotherapy.

## Introduction

Oral leukoplakia is defined as a “white plaque of questionable risk having excluded other (known) diseases or disorders that carry no increased risk for cancer” (1, 2). The prevalence increases with age and globally ranges from 1% to 5% in the general population (3, 4). While the vast majority of oral leukoplakia will remain stable, annual malignant transformation rates in the United States approach 3% (5). Several factors impact the risk of malignant change: histologic degree of dysplasia, location in the oral cavity, type of leukoplakia, size, presence of erythema (erythroleukoplakia), and tobacco use history (6, 7). Unlike localized leukoplakia lesions, proliferative (verrucous) leukoplakia (PVL or proliferative leukoplakia) describes a distinct subgroup of aggressive leukoplakia with a high rate of malignant transformation (approaching 10% per year; ref. 8) – characterized by nonhomogeneous or verrucous lesions typically involving multiple oral mucosal subsites (9).

Beyond clinically distinct forms of leukoplakia, histologic characterization or the presence of epithelial dysplasia is associated with step-wise progression to oral cancer (10). There is also an entity known as hyperkeratosis with minimal to no cytologic atypia [keratosis of unknown significance (KUS) or hyperkeratosis, nonreactive (HkNR); ref. 11], which is common among leukoplakia biopsies and may also harbor malignant potential (12); as prior molecular studies suggest genetic overlap between KUS and dysplasia includes frequent alterations in *TP53* and *KMT2C* (13). In addition, several preclinical studies have reported increased expression of programmed cell death receptor 1 (PD-1) ligand (PD-L1), on leukoplakic lesions with higher degrees of dysplasia (14–16), suggesting that immune escape may serve as a key mechanism for malignant transformation to oral carcinoma. To that end, we are currently accruing to a clinical trial of the PD-1 inhibitor nivolumab as preventative immunotherapy in high-risk oral leukoplakia, namely PVL or proliferative leukoplakia (NCT03692325).

As a precursor to our actively enrolling preventative immunotherapy trial, we employed comprehensive immunophenotyping of a cohort of localized leukoplakia and proliferative leukoplakia specimens in this study. We sought to determine tissue resident immune cell activation and spatial distribution, integrated with immunologic gene expression profiling (GEP), to characterize the complex oral immune microenvironment of each of these entities and to further nominate therapeutic targets aimed at oral cancer immunoprevention.

## Materials and Methods

### Study Participants and Disease Outcomes

Patients were retrospectively identified from an existing fully clinically annotated dataset of 149 patients with oral leukoplakia managed jointly at Dana-Farber Cancer Institute (DFCI, Boston, MA) and the Brigham & Women's Hospital (BWH, Boston, MA) Division of Oral Medicine and Dentistry. Following Institutional Review Board (IRB) approval (DF/HCC protocol# 19-765), patient demographics, clinical characteristics, and survival outcomes were recorded. Patient oral leukoplakia tissue samples were retrieved from archives and their pathologic diagnosis (ranging from KUS to varying degrees of dysplasia: mild, moderate, or severe) and clinical phenotype (localized leukoplakia vs. proliferative leukoplakia) verified by an expert oral pathologist (S.-B. Woo); previously having been interpreted by one of two head and neck pathology faculty members (any discordance was reviewed in multidisciplinary oral pathology conference). Date of first diagnosis of oral leukoplakia, number of oral biopsies obtained in follow-up over time, and time to a first head and neck cancer diagnosis (along with pathologic staging) were summarized. Inclusion in this study required sufficient mRNA isolated from oral leukoplakia tissue for immunologic GEP analysis. Research was conducted in accordance with the U.S. Common Rule and informed consent waived by the IRB due to limited risk to study participants.

### NanoString Immune Gene Expression Analysis

RNA from each oral leukoplakia specimen was isolated from cores punched from areas of epithelial dysplasia (High Pure FFPET RNA Isolation Kit, Roche Diagnostics) marked on formalin-fixed paraffin-embedded (FFPE) tissue slides and quantified (NanoDrop Products; Thermo Scientific). Isolated RNA was run on Bioanalyzer to obtain DV200 values (fragment above 200 bp) using Agilent RNA 6000 Pico Kit (Agilent Technologies). A minimum of 100 ng of isolated RNA with greater than DV200 per sample was loaded and run on the HuV1_CancerImmu_v1_1 NanoString platform for analysis of the NanoString PanCancer Immune Profiling Panel (PCI), as described previously (17). The nCounter Analysis System is based on a novel digital color-coded barcode technology and PCI provides a highly multiplexed GEP designed to quantitate 770 genes that fall into four functional categories (infiltrating immune cell types, immunologic function, tumor-specific antigens, and housekeeping genes). A version of the Tumor Inflammation Signature (TIS; ref. 18), an 18-gene signature that measures a preexisting but suppressed adaptive immune response within various cancers was utilized. Significantly, differentially expressed genes and additional signatures were computed using NanoString nSolver Advanced Analysis Module after normalization to default housekeeping genes with at least 100 counts.

### Multiplexed Immunofluorescence Staining with Digital Image Analysis

Multiplexed immunofluorescence (MIF) was performed on 4-μm–thick, FFPE whole tissue sections from oral leukoplakia specimens using a BOND RX Automated Stainer (Leica Biosystems) as described previously (19, 20). Briefly, FFPE tissue slides were baked for 3 hours at 60°C and loaded into the BOND RX. Slides were deparaffinized with BOND Dewax Solution (Leica Biosystems) and rehydrated through a graded series of ethanol and deionized water washes. BOND Epitope Retrieval Solution 1, pH 6.0 (Leica Biosystems) was used for antigen retrieval for 10 minutes at 98°C. Slides were serially incubated with primary antibodies at room temperature for 30 minutes as detailed in Supplementary Table S1, followed by anti-mouse plus anti-rabbit Opal Polymer Horseradish Peroxidase (Opal Polymer HRP Ms + Rb, Akoya Biosciences, catalog no. ARH1001EA) as a secondary label for 10 minutes. Opal Fluorophore Reagents (Akoya) were applied for 10-minute incubations to fluorescently label the antibody complexes. Finally, slides were incubated with Spectral DAPI solution (Akoya) for 10 minutes, air dried, and mounted with Prolong Diamond Anti-fade mounting medium (Life Technologies, catalog no. P36965) and stored in a light-proof box at 4°C prior to imaging. Image acquisition at 20× resolution was performed using the Vectra Polaris multispectral imaging platform (Vectra Polaris, Akoya Biosciences). Two geographically distinct regions are selected for each dysplastic tissue slide to best represent the overall tissue microenvironment. Three to six regions of interest (ROI) were then selected for analysis using Phenochart 1.0.12. Areas without dysplasia or residual normal mucosa were excluded. After ROI annotation, fields of view are spectrally unmixed and analyzed by supervised machine learning algorithms within inForm 2.4.8 (Akoya Biosciences). Each cell phenotype–specific algorithm is based upon an iterative training or test process, whereby a small number of cells (training phase, typically 20–30 cells) are manually selected as being most representative of each phenotype of interest and the algorithm then predicts the phenotype for all remaining cells (testing phase; ref. 21). The pathologist (S.J. Rodig) can over-rule the decisions made by the software to improve accuracy, until phenotyping is optimized. Thresholds for "positive" staining and the accuracy of phenotypic algorithms were then optimized and confirmed by the pathologist (S.J. Rodig) for each case. Quantities, spatial attributes, and graphical abstractions were then generated from inForm images based on an image analysis pipeline (19, 20). Physical contacts between dysplastic cells and neighboring immune cells were determined on the basis of membrane maps produced by inForm; and the percentage of each cell phenotype among cells was calculated within the vicinity of dysplastic cells. Mean count density was determined as the average of cell counts obtained among multiple imaging frames. Combined positive score (CPS) and dysplastic (replacing the word “tumor”) epithelium proportion score (TPS) were defined as all PD-L1–positive cells (CD8^+^, dysplastic, or other immune cells) or all PD-L1–positive dysplastic epithelial cells each divided by any dysplastic epithelial cells (stained using cytokeratin), respectively, across processed digital slide images. Four known head and neck squamous cell carcinoma cases served as internal controls.

### Statistical Analysis

Descriptive statistics were used to summarize patient demographics. Fisher exact test (categorical variables) and a Mann–Whitney test (continuous variables) was used to characterize similarity between localized leukoplakia and proliferative leukoplakia subgroups with respect to clinical features. Overall survival (OS) was determined from the date of first oral leukoplakia diagnosis to death from any cause, while cancer-free survival (CFS) was determined from the date of first oral leukoplakia diagnosis to the diagnosis of first invasive squamous cell carcinoma of the oral cavity or death, whichever occurred first, or censored at last known follow-up (using Kaplan–Meier estimates and log-rank testing to compare localized leukoplakia and proliferative leukoplakia subgroups). Oral leukoplakia tissue utilized on study was that obtained at or near initial diagnosis, not subsequent oral leukoplakia biopsies, and prior to any topical mucosal therapy application. Associations between immune cell infiltration and GEPs with clinical characteristics was made under a general linear model (nonparametric) with Student *t* tests (Mann–Whitney test), ANOVA (Kruskal–Wallis test), and Pearson correlation coefficients, as appropriate. Clinicopathologic features, immunophenotypes, and gene signatures were correlated with survival outcomes and risk of recurrence (Kaplan–Meier method, Cox proportional hazards modeling, binary logistic regression modeling). Statistical analyses utilized STATA version 14.2. A nominal *P* < 0.05 was used to denote statistical significance. For each GEP, the Benjamini–Yekutieli procedure was used to control the false discovery rate under arbitrary dependence assumptions.

### Data Availability

The data generated in this study are available upon request from the corresponding author. Multiplexed GEP profiling data generated from RNA (NanoString) was deposited in the public repository Gene Expression Omnibus (GEO; accession number GSE184944).

## Results

### Clinical Features and Survival Outcomes

**FIGURE 1:**
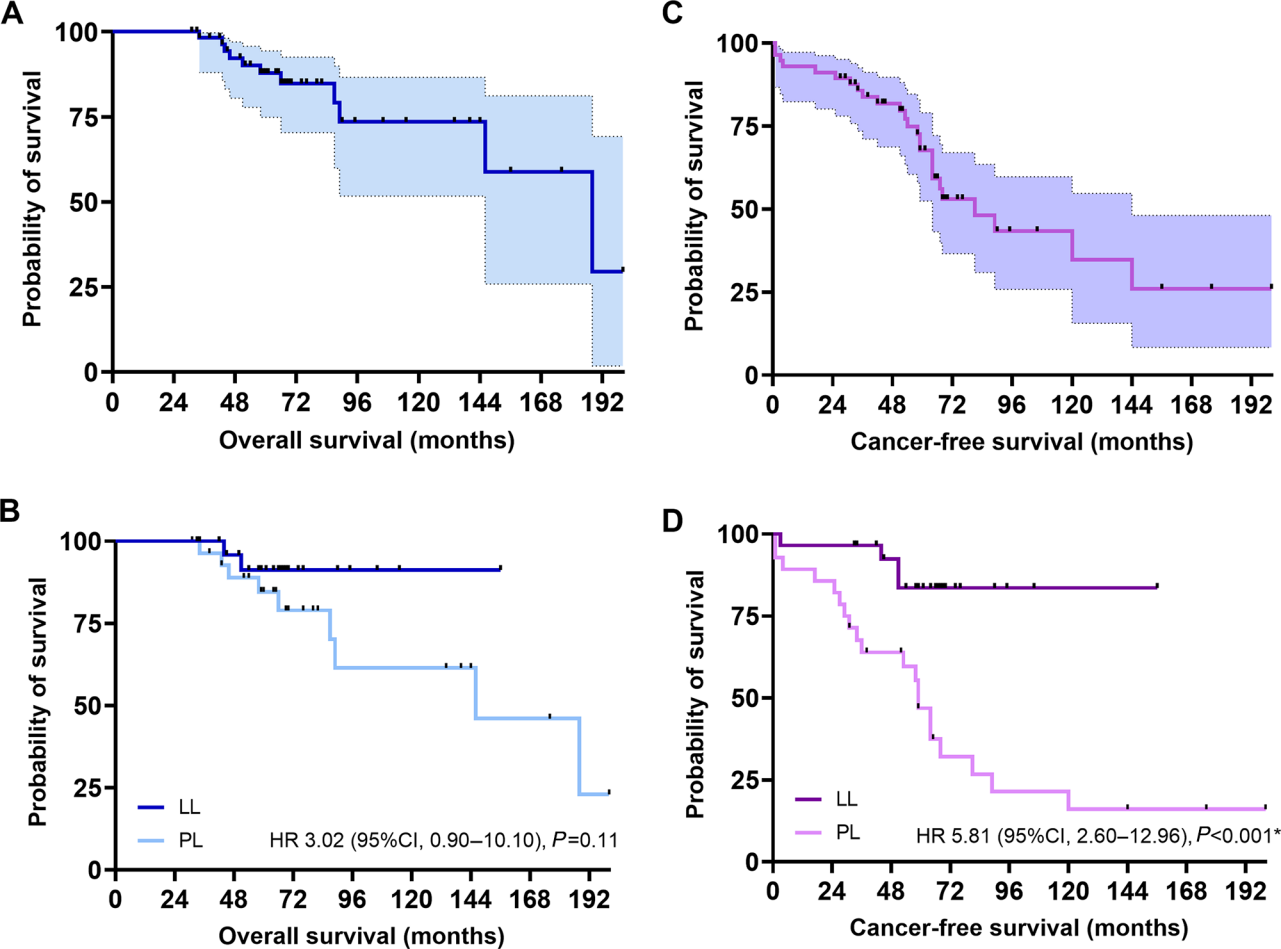
Overall survival among the total population (**A**) and among patients with localized leukoplakia (LL; **B**) and proliferative leukoplakia (PL). CFS among the total population (**C**) and among patients with localized leukoplakia and proliferative leukoplakia (**D**). Kaplan–Meier method, log-rank testing. *, *P* < 0.05.

From our initial retrospective single institution cohort of 149 patients first diagnosed with an oral leukoplakia between 2000 and 2018, 78 had localized leukoplakia and 71 had proliferative leukoplakia. Among 58 randomly selected patients with available (nonexhausted) tissue samples the two prespecified groups of localized leukoplakia (*n* = 29) and proliferative leukoplakia (*n* = 29) were balanced in terms of baseline characteristics such as age, gender, smoking history, oral cavity subsite, and pathologic diagnosis (Table 1). Seven of 29 (24%) patients with proliferative leukoplakia had received a median of 1 (range: 1–3) topical oral mucosal therapies (steroid elixir or rinse, topical immunomodulating agent) for their oral leukoplakia prior to a first cancer diagnosis. There were more cancer events in the proliferative leukoplakia (17, 59%) versus the localized leukoplakia group (2, 7%) during study follow-up, and proliferative leukoplakia cancers were more often advanced stage at diagnosis (8, 28% stage III–IVA/B). With a median follow-up of 65 months, there was 8 versus 1 death in the proliferative leukoplakia versus localized leukoplakia groups, respectively. Median OS was not reached (NR) in the localized leukoplakia group versus 146 months in the proliferative leukoplakia group [HR, 0.33; 95% confidence interval (CI), 0.09–1.10; *P* = 0.11]; 5-year OS estimate: 91.3% vs. 84.6% and 10-year OS estimate: 91.3% vs. 61.4%, respectively (Fig. 1). Median CFS was superior in the localized leukoplakia group (NR vs. 59 months; HR, 0.17; 95% CI, 0.07–0.38; *P* < 0.001), with a 2-year CFS of 96.5% versus 82.1% and 5-year CFS of 83.6% versus 46.8% in the localized leukoplakia versus proliferative leukoplakia group. CFS did not appear to differ when considering patient age at diagnosis (HR, 0.54; *P* = 0.18), gender (HR, 1.00; *P* = 0.68), or smoking history (HR, 0.63; *P* = 0.75) in the multivariable model. Similarly, primary site of oral leukoplakia (HR, 0.73; *P* = 0.72) and degree of epithelial dysplasia (HR, 0.98; *P* = 0.95) did not predict CFS. Only a clinical diagnosis of proliferative leukoplakia was associated with significantly decreased CFS (HR, 11.25; 95% CI, 2.60–48.72; *P* < 0.01; Supplementary Table S2).

**TABLE 1:** Baseline patient characteristics

Characteristics	All (%)^a^, *N* = 58	LL, *n* = 29	PL, *n* = 29
Age at diagnosis, years	64 (27–79)	66 (27–79)	63 (35–76)
Gender Male Female	26 (45) 32 (55)	14 (48) 15 (52)	12 (41) 17 (59)
Smoking history Never Former smoker (>10 pack-years) Current smoker	26 (45) 28 (48) 4 (7)	11 (38) 15 (52) 3 (10)	15 (52) 13 (45) 1 (3)
Primary site of disease Buccal mucosa Oral tongue Maxillary alveolar gingiva Mandibular alveolar gingiva Soft palate Multifocal	9 (16) 26 (45) 2 (3) 4 (7) 3 (5) 14 (24)	6 (21) 19 (66) 0 2 (7) 2 (7) 0	4 (14) 5 (17) 3 (10) 2 (7) 1 (3) 14 (48)
Pathologic diagnosis on biopsy^b^ Atypical epithelial proliferation (AEP) Keratosis of undetermined significance (KUS) Mild dysplasia Moderate dysplasia Severe dysplasia	4 (7) 32 (55) 12 (21) 8 (14) 2 (3)	1 (3) 17 (59) 6 (21) 3 (10) 2 (7)	3 (10) 15 (52) 6 (21) 5 (17) 0
No. of biopsies obtained in follow-up	2 (1–7)	2 (1–4)	3 (1–7)
No. diagnosed with oral cavity SCC in follow-up	19 (33)	2 (7)	17 (59)
Time to first oral cavity SCC diagnosis	31 (<1–120)	27 (3–51)	31 (<1–120)
Pathologic stage of first head and neck cancer diagnosis^c^ pT1-2N0 (stage I, II) pT1-4N1-2b (stage III, IVA/B)	11 (58) 8 (42)	2 (11) 0	9 (31) 8 (28)

NOTE: LL, localized leukoplakia; PL, proliferative leukoplakia; SCC, squamous cell carcinoma.

^a^Values are numbers and percentages, except: age at diagnosis, no. of biopsies obtained, and time to first head and neck cancer diagnosis; noted as median and range in parentheses.

^b^First biopsy obtained as part of study entry.

^c^American Joint Committee on Cancer (AJCC) 2017 8th edition staging.

### Proliferative Leukoplakia Phenotype Associated with a Cytotoxic T-Cell Signature

**FIGURE 2:**
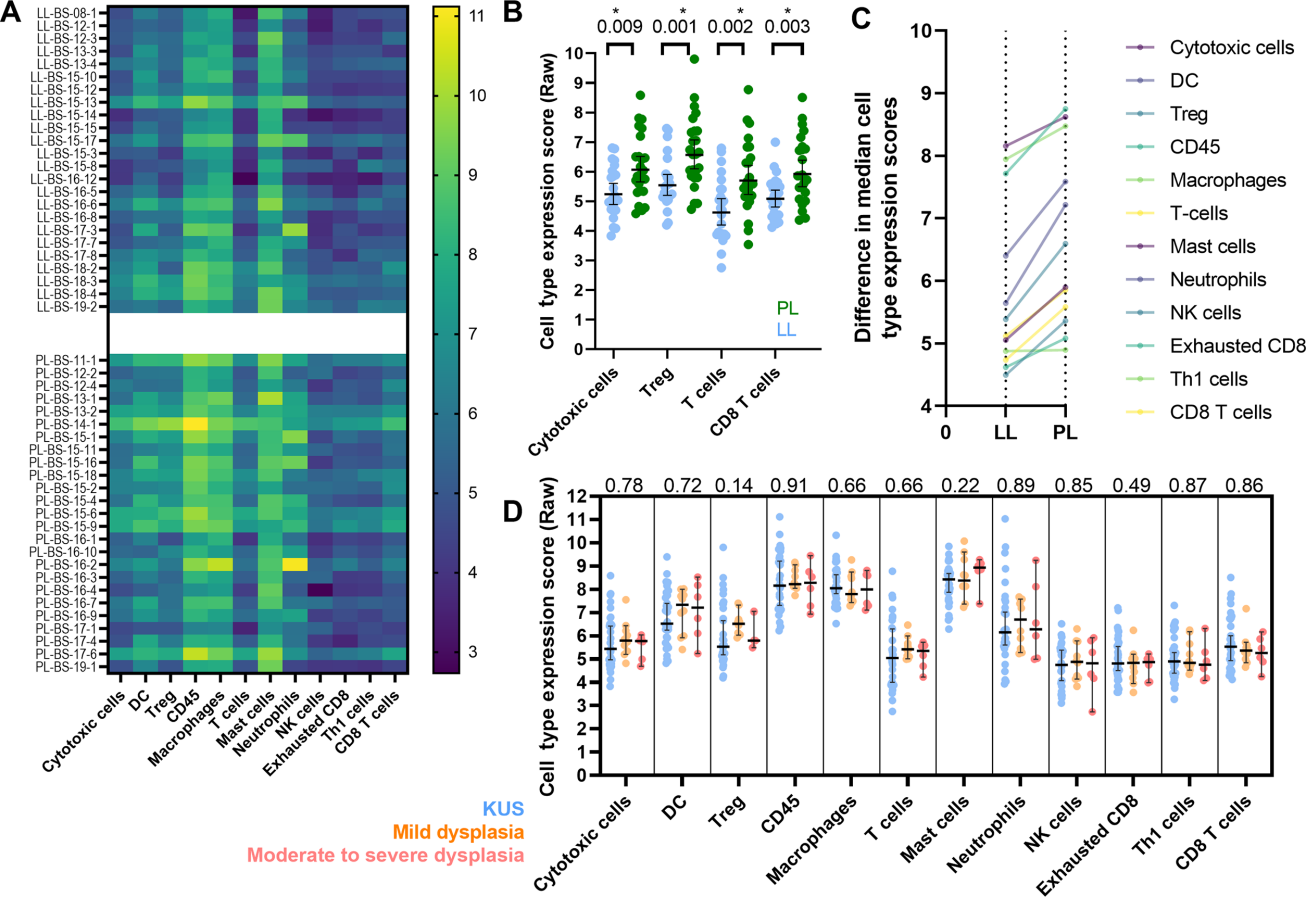
**A,** Heat map comparing RNA expression of immunoregulatory genes among localized leukoplakia (LL; top) and proliferative leukoplakia (PL; ; bottom) samples with protein expression grouped by immune cell type of importance (in each column). Cell type raw scoring (dark to light) indicates increasing absolute degree of protein RNA expression. **B,** Protein RNA expression of all immunomodulatory genes grouped by immune cell of interest and compared between localized leukoplakia and proliferative leukoplakia samples (Mann–Whitney test). Median immune cell expression levels compared between localized leukoplakia and proliferative leukoplakia (higher slope equating to greater difference; **C**) and compared by degree of epithelial dysplasia histologically (Kruskal—Wallis test; **D**). *, *P* < 0.05; two-sided.

**FIGURE 3:**
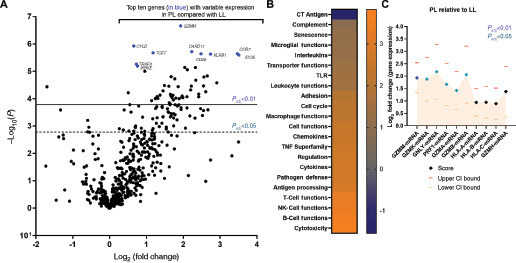
**A,** Volcano plot among all cohort samples showing the log_2_ fold change in mRNA expression at the individual protein level plotted against adjusted −log_10_*P* value (degree of significance) among proliferative leukoplakia (PL) samples compared with localized leukoplakia (LL) samples. The top 10 genes with variable expression at higher significance are identified in blue and identified by name. *, *P* < 0.05; adjusted using the Benjamini–Yekutieli procedure (false discovery rate). **B,** Heat map of global significance scores compared among proliferative leukoplakia and localized leukoplakia (lighter color means higher positive scores) among genes/mRNA organized by immunologic function or pathway. **C,** Log_2_ fold change in mRNA expression scores among cytotoxicity genes in the immune panel among proliferative leukoplakia relative to localized leukoplakia samples. Upper and lower CI values are shown. *, *P* < 0.05 (adjusted) denoted by colored diamonds.

We first compared immune cell type RNA expression profiles for all localized leukoplakia and proliferative leukoplakia samples (Fig. 2). While dysplasia microenvironment (DME) immune cell composition was largely similar, there were important differences: proliferative leukoplakia samples demonstrated greater cell type expression scores profiling CD8^+^ T cells, cytotoxic T cells, and T regulatory cells (Treg; all *P* < 0.01). When comparing immune cell type expression profiles among all samples based on their histologic characterization (KUS; mild, moderate, or severe dysplasia) irrespective of their localized leukoplakia or proliferative leukoplakia phenotype, DME composition was similar. In addition, when considering clinical parameters such as younger age at diagnosis (<45 years old), gender, and smoking history among the cohort, DME composition was similar (all *P* < 0.05). However, only an increased abundance of Tregs predicted first oral cancer progression (OR, 2.30; *P* < 0.01; Supplementary Table S3). Next, we sought to interrogate which cytotoxicity genes accounted for immune cell type profiling differences among the localized leukoplakia and proliferative leukoplakia subgroups. Gene set analysis (summarizing changes in regulation within each defined gene set) aimed at comparing individual immunologic mRNA expression scores among localized leukoplakia and proliferative leukoplakia samples clarified that granzyme-M (GZMM) was the most significantly differentially expressed gene favoring the proliferative leukoplakia subgroup (log_2_ fold change, 1.93; *P*_adj_ < 0.001) (Fig. 3). In addition, *CYLD*, *CARD11*, *TCF7*, *CCR7*, *KLRB1*, *CD28*, and *ICOS* were other significantly highly expressed genes among the proliferative leukoplakia subgroup (log_2_ fold changes, 0.65–3.51; all *P*_adj_ = 0.001). Binary logistic regression modeling suggested greater log_2_ expression among CYLD (OR = 9.01) and TCF7 (OR = 6.29) predicted risk of progression to cancer regardless of localized leukoplakia or PL phenotype (both *P* = 0.01 or less; Supplementary Table S3). Global significance scores (GSS) were determined to measure the overall differential expression of selected genes relative to localized leukoplakia or proliferative leukoplakia phenotype ignoring whether genes were up- or downregulated. GSS favoring proliferative leukoplakia were highest among cytotoxicity (3.82), B-cell function (3.56), and NK-cell function (3.34) pathways. When interrogating the 10 genes that comprised the cytotoxicity pathway, GZMM along with GZMK, GNLY, PRF1, GZMA, and GZMB were all significantly differentially expressed among proliferative leukoplakia samples (all *P*_adj_ < 0.05).

### PD-1–Expressing CD8^+^ T Cells and Tregs Colocalize to the Dysplasia–Stromal Interface in Proliferative Leukoplakia

**FIGURE 4:**
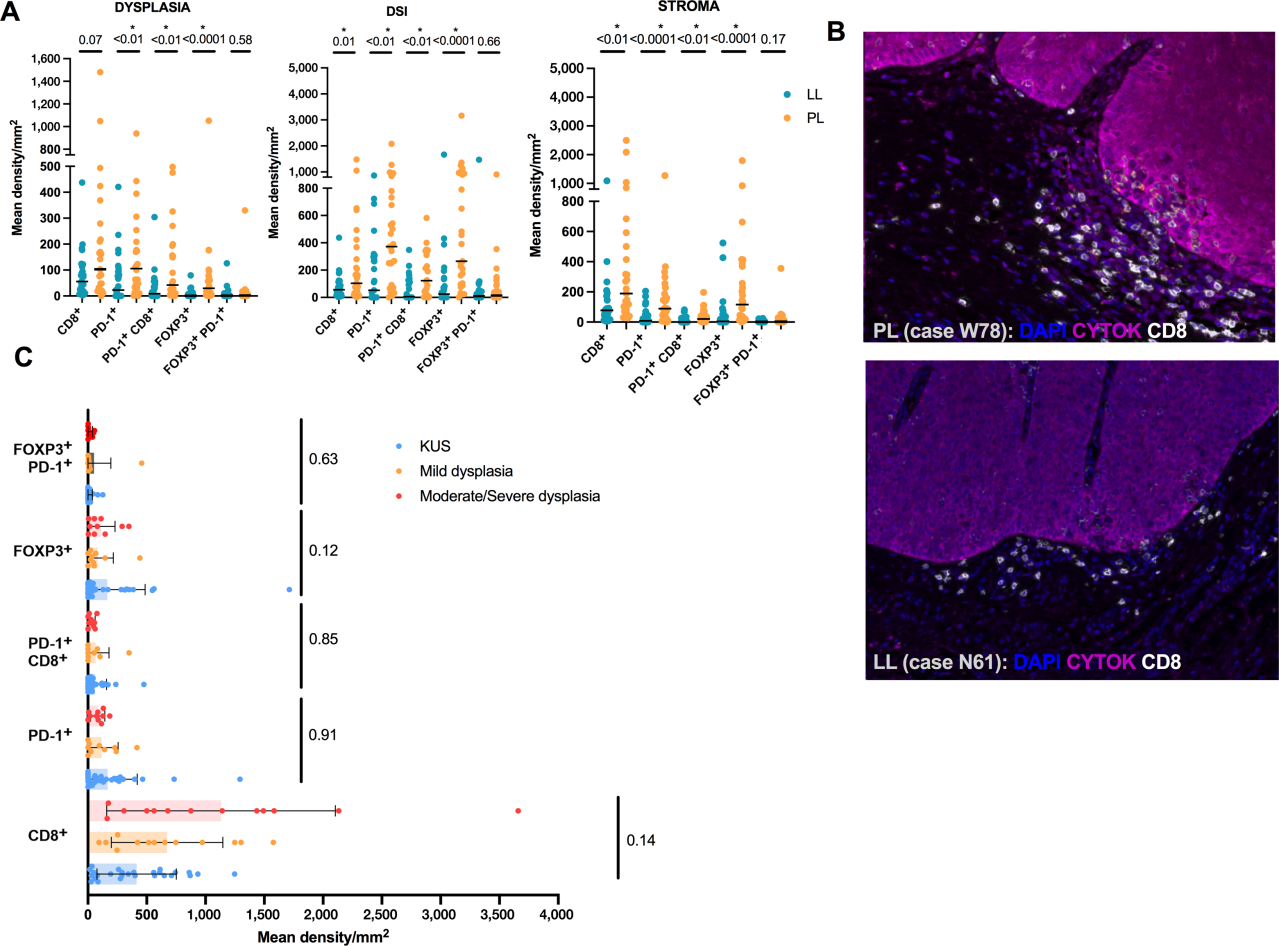
**A,** Box plots showing mean density (in mm^2^) of immune cell populations separated by localized leukoplakia (LL) and proliferative leukoplakia (PL) subgroups where each column shows individual values and mean is denoted by the solid line. Graphs plot the immune cell content within the dysplastic tissue, at the DSI, and within the stroma separately. *, *P* < 0.05, Mann–Whitney; two-sided. **B,** MIF imaging showing spatial representation of immune cells along the DSI within two cases (PL, W78, and LL, N61). DAPI, stains live cells; CYTOK, cytokeratin. **C,** Box plot showing mean density (in mm^2^) of immune cell populations separated by degree of dysplasia histologically regardless of localized leukoplakia or proliferative leukoplakia clinical phenotype. *, *P* < 0.05, Kruskal–Wallis; two-sided.

To complement our immunologic mRNA expression profiling across localized leukoplakia and proliferative leukoplakia subgroups, we next sought to understand spatial or geographic localization patterns of immune cells within the dysplastic or premalignant epithelium (D), the stroma (S), and at the dysplasia–stromal interface (DSI) measured within 40 μm of the actual dysplasia–stromal border. Fifty-five of 58 samples passed quality control metrics for MIF analysis. The mean abundance of CD8^+^ T cells was significantly increased at the DSI (869 vs. 415 cells/mm^2^, *P* = 0.02) when comparing proliferative leukoplakia and localized leukoplakia subgroups, but overall median density (cells/mm^2^) was high among all cohort samples (range: 9–3659). We observed significantly increased mean density (cells/mm^2^) of all PD-1^+^ T cells and PD-1^+^ CD8^+^ T cells (D, 42.3 vs. 8.8; S, 21 vs. 0; DSI, 122.8 vs. 5.4) within the dysplastic epithelium, the stroma, and at the DSI (all *P* < 0.01) among proliferative leukoplakia tissues compared with localized leukoplakia. In addition, a significantly greater abundance (cells/mm^2^) of FOXP3^+^ Tregs were observed among proliferative leukoplakia tissue samples at the dysplastic epithelium, the DSI, and in the stroma (D, 29.8 vs. 0.8; DSI, 265.7 vs. 24.2; S, 115.8 vs. 4.3; all *P* < 0.001). Figure 4 visually illustrates examples of CD8^+^ T-cell localization to the DSI among proliferative leukoplakia and localized leukoplakia tissues, highlighting the significant increase in T-cell abundance among high-risk proliferative leukoplakia cases when utilizing MIF digital overlay. When considering localization of PD-1^+^ T cells and Tregs among tissues in the cohort based on histology patterns, findings were similar across tissue regions (D, DSI, or S) regardless of degree of dysplasia; although the mean density (cells/mm^2^) of CD8^+^ T cells tended to be higher among moderate-to-severely dysplastic tissues (1131 vs. 673 for mild dysplasia, and 414 for KUS; *P* = 0.143).

### Stromal PD-L1 Overexpression within Proliferative Leukoplakia

Having characterized the geospatial distribution of key immune cells within proliferative leukoplakia and localized leukoplakia samples at the DSI, we next aimed to quantitate and characterize PD-L1 expression patterns throughout these tissues. Broadly, both PD-L1 expression as defined by CPS (dysplastic epithelium and other immune cells) or TPS (epithelial dysplastic cells) was significantly increased among proliferative leukoplakia samples compared with localized leukoplakia (CPS, 2.45 vs. 0; localized leukoplakia: 0.1 vs. 0; both *P* < 0.0001), but notably values for both were frequently measured at 1 or less (78.1%). When accounting for PD-L1 CPS expression across the dysplastic and stromal tissue area, PD-L1 expression was significantly higher at all subsites (D, DSI, and S) among proliferative leukoplakia samples (D, 0.41 vs. 0; DSI, 3.99 vs. 0; S, 362 vs. 0; all *P* < 0.0001). However, when evaluating PD-L1 CPS across all samples by degree of dysplasia assessed histologically, regardless of leukoplakia phenotype, expression levels were similar (*P* = 0.94).

**FIGURE 5:**
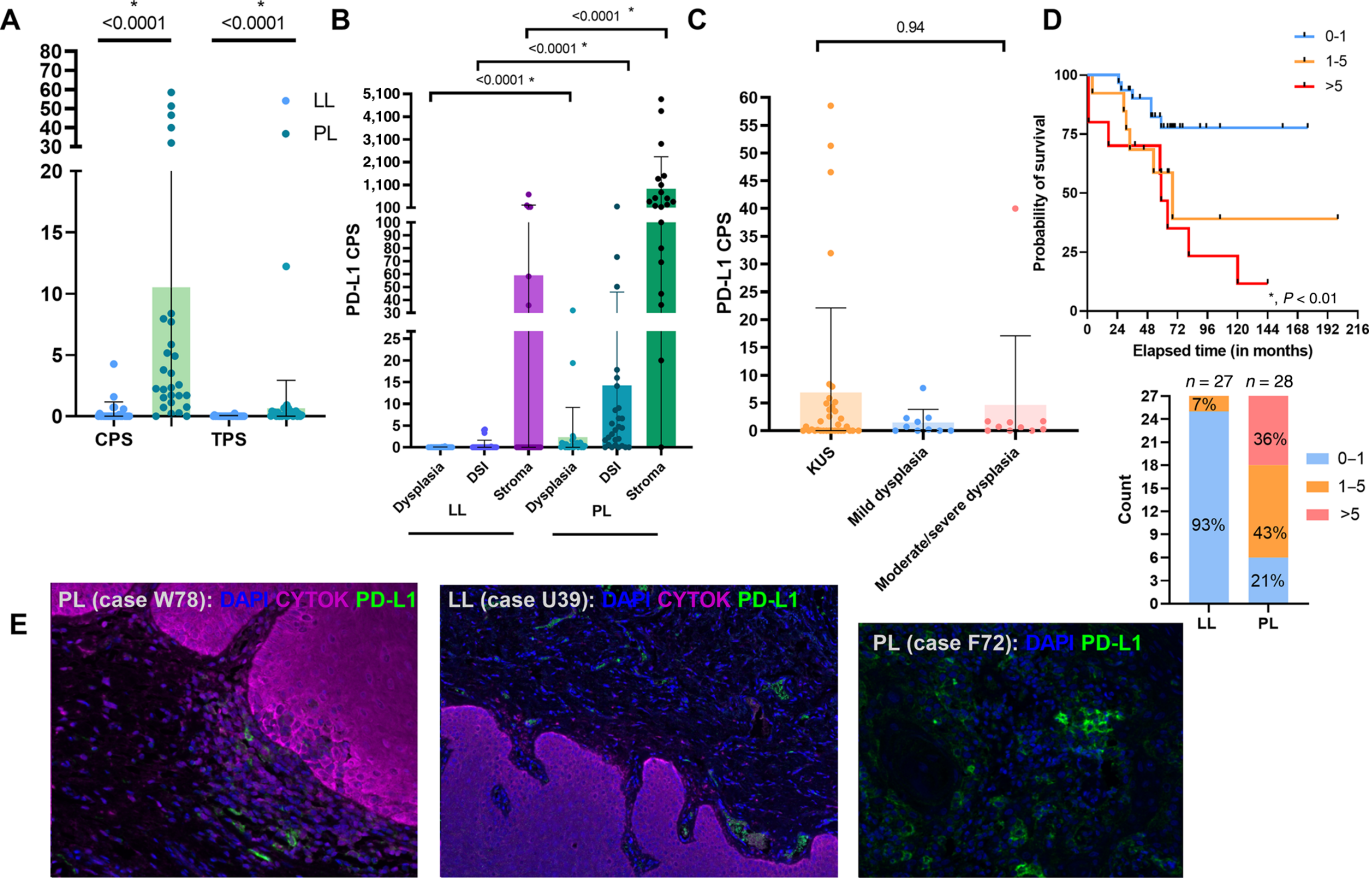
**A,** CPS and TPS characterizing PD-L1 expression compared among localized leukoplakia (LL) and proliferative leukoplakia (PL). *, *P* < 0.05, Mann–Whitney test; two-sided. **B,** PD-L1 CPS scores and mean values compared between differing regions of the DSI and within dysplastic and stromal tissues for both localized leukoplakia and proliferative leukoplakia samples. *, *P* < 0.05, Kruskall–Wallis test; two-sided. **C,** PD-L1 CPS values and mean scores compared by degree of histologic dysplasia. *, *P* < 0.05, Kruskall–Wallis test, two-sided. **D,** Kaplan–Meier estimate of CFS separated by PD-L1 CPS scores among pooled localized leukoplakia and proliferative leukoplakia samples. *, *P* < 0.05, log-rank testing; and bar graph showing PD-L1 CPS total counts among localized leukoplakia and proliferative leukoplakia samples. **E,** MIF imaging highlighting PD-L1 staining and spatial arrangement near the DSI among representative proliferative leukoplakia and localized leukoplakia samples. CYTOK, cytokeratin; DAPI, stains live cells.

Using previously reported PD-L1 CPS cutoffs of 0–1, 1–5, and greater than 5, we observed a significantly improved CFS at low PD-L1 expression levels (0–1; 5-year CFS estimates: 79% vs. 62% vs. 37%, respectively; *P* < 0.01); recognizing that 26 of 27 (93%) of all localized leukoplakia tissue samples demonstrated such low PD-L1 CPS expression levels compared with 6 of 28 (21%) of proliferative leukoplakia tissue samples. Figure 5 visually illustrates examples of PD-L1^+^ immune cells localized to the stroma and DSI among proliferative leukoplakia and localized leukoplakia tissues, highlighting the significant increase in PD-L1^+^ immune cell abundance among high-risk proliferative leukoplakia cases when compared with localized leukoplakia utilizing MIF digital overlay.

## Discussion

Distinguishing localized leukoplakia (localized leukoplakia) from the high-risk entity of proliferative leukoplakia (proliferative leukoplakia) has important implications with regards to surveillance and management. Proliferative leukoplakia is typically characterized clinically by a verrucous appearance, either at more than two noncontiguous oral cavity subsites or extensively at one or contiguous sites, and histologically demonstrates epithelial hyperkeratosis or verrucous hyperplasia (9). Proliferative leukoplakia is often difficult to treat based on its multifocality and typically large size of single-site lesions, while malignant transformation rates to oral carcinoma approach 70% in some series (22, 23). This is in comparison to the more indolent localized leukoplakia where transformation rates rarely exceed 15% (5). As expected, we demonstrate significantly decreased CFS when comparing proliferative leukoplakia to localized leukoplakia subgroups with 2-year and 5-year CFS estimates among patients with proliferative leukoplakia at 82.1% and 46.8%, respectively, with an 8-fold increase in cancer events (17 vs. 2) among the proliferative leukoplakia group at median follow-up of over 5 years. Clinicopathologic features such as smoking history and degree of epithelial dysplasia (on representative biopsies) did not significantly impact CFS; in fact, only the clinical diagnosis of proliferative leukoplakia portended worse outcomes on multivariable analysis. While there was no significant difference in OS between proliferative leukoplakia and localized leukoplakia subgroups, survival was inferior among patients with proliferative leukoplakia with a 5-year OS estimate of 84.6%. One could argue that close and frequent surveillance (every 3 months) by experts in an academic oral medicine and head and neck oncology program could have detected cancer events sooner thus yielding earlier treatment and a survival impact for patients with proliferative leukoplakia. However, 8 of 17 (47%) patients with proliferative leukoplakia who developed oral carcinoma on study were diagnosed with pathologic stage III, IVA, or IVB disease.

Our group previously published on the genomic characterization of oral precancerous lesions showing that early precursor lesions like KUS share somatic alterations with epithelial dysplastic lesions with frequent mutations in *KMT2C* and *TP53* (13). While only 6 of 20 (30%) patients in that molecular study were characterized as having proliferative leukoplakia, other studies aimed at genotyping the proliferative leukoplakia subgroup have nominated alterations in *TP53* and *CDKN2A* as important (24, 25). Beyond somatic alterations in tumor suppressor genes, immunologic dysregulation or imbalance does seem to contribute to malignant transformation risk among oral precancerous lesions (26). It is posited that the stepwise progression from epithelial dysplasia to frank oral squamous cell carcinoma relies on immune escape mechanisms within the microenvironment.

Several recent studies have aimed to broadly characterize the immunologic landscape of oral leukoplakia. Among a cohort of 80 oral leukoplakias evaluated by IHC for immunomodulatory mediators, increased expression of HLA-G/E, IL10, TGFβ2/3 was noted when compared with oral carcinoma samples (27). Subsequently, several oral leukoplakia cohorts have demonstrated upregulation of PD-L1 compared with normal mucosal tissues that often correlates with CD8^+^ T-cell infiltration (16, 28, 29) suggesting a role for evasion of the host immune system as part of malignant transformation. To that end, Ries and colleagues further showed that PD-1/PD-L1 expression in the epithelial and subepithelial layer of oral leukoplakia was increased in tissues where malignant transformation was later observed (14). This study builds on these findings, employing more comprehensive immune characterization methods with mRNA expression profiling and MIF aimed at immunologically defining proliferative leukoplakia. Among proliferative leukoplakia cases, higher PD-L1 CPS predicted higher median PD-1+ CD8^+^ T-cell abundance (PD-L1 CPS > 5, 118.3 cells/mm^2^; CPS 1–5, 43.5 cells/mm^2^; CPS < 1, 16.8 cells/mm^2^).

When considering immune cell types in the DME, CD8^+^ T cells, and cytotoxic T cells are increased among proliferative leukoplakia samples when compared with localized leukoplakia, while quantitative and spatial techniques clarify this occurs across all regions of the stroma and dysplastic tissue with the highest mean density of PD-1^+^ CD8^+^ T cells and Tregs homing to the DSI. This fits with the hypothesis that PD-1–overexpressing cytotoxic T cells migrate to the epithelial tissue border where interactions with dysplastic tissue expressing PD-L1 occurs, permitting immune checkpoint interaction and immune evasion to promote carcinogenesis. Similarly, an abundance of Tregs at the DSI among proliferative leukoplakia samples may promote tumor progression by limiting antidysplasia immunity (30). Others have shown that PD-1^+^ Tregs have the potential to proliferate and trigger immunosuppressive activity in the tumor microenvironment if exposed to PD-1 blockade (leading to clinical hyperprogression of disease; ref. 31).

We did not observe the same increase in mean cell density among cytotoxic or other immune cells when separating dysplastic tissue samples by degree of dysplasia in contrast to prior reports (28, 29). This difference could be explained by the inclusion of oral leukoplakia with specific phenotypes of interest (localized leukoplakia and proliferative leukoplakia) in this study; also, our methods involved gene expression profiling and automated digital capture compared with other studies relying solely on traditional IHC. Our findings suggest that clinical phenotype rather than degree of epithelial dysplasia may more often immunologically distinguish proliferative leukoplakia.

The proliferative leukoplakia subgroup had a more prominent cytotoxic T-cell signature as compared with localized leukoplakia, with differential upregulation of key genes important for regulating cytotoxicity function, namely granzymes. GZMM is one of many serine proteases housed in granules released from cytotoxic lymphocytes which later enter tumor cells via perforin to activate cell death pathways (32). However, IFNγ expression was significantly lower among the proliferative leukoplakia subgroup (log_2_ fold change −1.39, *P*_adj_ < 0.05). Another gene of interest with overexpression among proliferative leukoplakia samples was inducible T-cell costimulator (ICOS), which codes for a T-cell costimulatory receptor that promotes T-cell proliferation, chemokines, and facilitates B-cell antibody secretion (33). A previously published immune-related gene signature (IRGS) comprised of 27 genes of prognostic importance among 770 patients with head and neck squamous cell carcinoma (HNSCC; The Cancer Genome Atlas, TCGA) also identified ICOS as important (34). The ICOS agonist feladilimab showed some encouraging activity and durable benefit in a single-arm, early-phase combination study with the PD-1 inhibitor pembrolizumab (INDUCE-1) among 34 patients treated for recurrent, metastatic HNSCC (35). But recently, the subsequent phase II INDUCE-3 trial comparing feladilimab or placebo plus pembrolizumab among patients with PD-L1^+^ HNSCC was terminated for futility (36). These data suggests an anti–PD-1/ICOS agonist combination may be of some benefit earlier in oral cancer disease natural history; perhaps for patients with high-risk proliferative leukoplakia to mitigate the risk of malignant transformation to oral carcinoma. Also notable was the overexpression of TCF7 among proliferative leukoplakia (log_2_ fold change 1.18; *P*_adj_ = 0.001). TCF7^+^ CD8^+^ T-cell frequency has been linked with tumor regression or checkpoint inhibitor response in melanoma (37). TCF7 and CYLD (a tumor suppressor) were both strong predictors of risk of progression to cancer in this study.

Using CPS to measure tumor and immune cell PD-L1 expression among recurrent or metastatic HNSCC demonstrates that >80% of cases express PD-L1 at a cutoff of 1 or greater by IHC, but recognizing that an estimated 40% of tumors have scores ≥20 (38). Studies evaluating epithelial and subepithelial PD-L1 expression in oral leukoplakias have yielded values of 0–10 by IHC scoring – overall lower than those observed in the recurrent, metastatic setting although these groups have not been uniformly compared (14). We observed similar findings of relatively low PD-L1 CPS values across our study samples (typically ranging from 1–5) except for higher expression among stromal proliferative leukoplakia tissue which may reflect honing during immune engagement. PD-L1–positive immune cells appear spatially enriched at the stroma and DSI among proliferative leukoplakia epithelial tissues, which may promote PD-1/PD-L1 axis interactions to enhance tumorigenesis. While rates of PD-L1 expression have been reported to correlate with more severe dysplasia among oral leukoplakias (16), we did not observe the same. PD-L1 expression seemed to correlate better with proliferative leukoplakia phenotype, rather than degree of dysplasia in this study. The discrepancy in this observation may reflect that we knowingly enriched for proliferative leukoplakia cases as compared with other studies; and it's worth noting the proliferative leukoplakia phenotype often yields histologic findings of KUS or hyperkeratosis (not frank dysplasia). Despite the correlation between PD-L1 expression and proliferative leukoplakia observed in this study, PD-L1 expression is predictive of PD-1 inhibitor response in advanced head and neck cancers (39). Again, we note that our exploratory method for PD-L1 quantitation was automated and digital, which may explain discordance with IHC or visual counting noted in prior studies; high resolution PD-L1 quantification should be validated in future studies. Our data provide a strong preclinical rationale for the use of PD-1/L1 blockade as oral preventative immunotherapy among patients with high-risk proliferative leukoplakia, which is currently being tested at our institution (NCT03692325) and elsewhere (NCT03603223, NCT04504552).

We acknowledge some limitations in this study. First, we randomly selected 58 cases (39%) with available tissue samples for immune profiling among a preexisting dataset of 149 patients; although we aimed to minimize selection bias and subgroups were well balanced. We acknowledge that pathologic characterization of high-risk precancerous lesions can be variable. In addition, NanoString reflects gene rather than protein expression levels. Although MIF assessment of immune subsets were consistent with our GEP data, specific immune cell phenotypes (e.g., activated CD8^+^ T cells) were not delineated from the MIF panel due to multiplex limitations. Finally, we immunologically profiled dysplastic epithelial tissues at a single timepoint among each case, and thus dynamic changes in the DME or sample heterogeneity must be considered when interpreting our results.

Our findings add to an important and evolving literature that suggest high-risk oral precancerous dysplasia may progress to malignancy in part due to local intra-oral immune dysregulation (40). While prior studies have suggested the immunologic cellular response may relate to the degree of dysplasia among oral leukoplakias (41, 42), we demonstrate that the proliferative leukoplakia phenotype is more often associated with a CD8^+^ cytotoxic T-cell infiltrate with granzyme overexpression that localizes throughout the DSI. A predominant portion of PD-L1^+^ immune cells appear enriched throughout the tissue stroma and DSI. While PD-L1 CPS scores are low among most oral leukoplakias, proliferative leukoplakia demonstrates higher relative PD-L1 scores which may predict benefit from PD-1/PD-L1 axis blockade alone or in combination with other novel immunotherapies. Actively enrolling trials are already investigating oral preventative immunotherapy among this critical population.

## Supplementary Material

Supplementary Table 1 Target antigens, antibody clones, dilution of markers, and antigen retrieval conditions used for multiplex immunofluorescence staining

Supplementary Table 2 Clinicopathologic predictors of cancer-free survival among patients with oral leukoplakia

Supplementary Table 3 Immunologic and molecular predictors of progression to cancer among oral leukoplakias
