# Correction: Comprehensive Immunoprofiling of High-risk Oral Proliferative and Localized Leukoplakia

**DOI:** 10.1158/2767-9764.CRC-22-0193

**Published:** 2022-05-19

**Authors:** Glenn J. Hanna, Alessandro Villa, Nikhil Mistry, Yonghui Jia, Charles T. Quinn, Madison M. Turner, Kristen D. Felt, Kathleen Pfaff, Robert I. Haddad, Ravindra Uppaluri, Scott J. Rodig, Sook-Bin Woo, Ann Marie Egloff, F. Stephen Hodi

In the original version of this article (1), the reported CD8^+^ T-cell quantifications in Fig. 4 were incorrect due to an error in data extraction. The CD8^+^ T-cell quantifications have been corrected throughout the article text and figure. In addition, the related statistical tests in Fig. 4A and 4C have been corrected accordingly. The latest HTML and PDF versions both reflect these corrections, which do not affect the main conclusions of this study. The authors regret this error.

## References

[1] Hanna GJ , Villa A, Mistry N, Jia Y, Quinn CT, Turner MM, . Comprehensive immunoprofiling of high-risk oral proliferative and localized leukoplakia. Cancer Res Commun 2021;1:30–40. 36860910 10.1158/2767-9764.CRC-21-0060 PMC9973379

