# Supplementary material for: Comprehensive Immunoprofiling of High-Risk Oral Proliferative and Localized Leukoplakia
Source: Cancer Res Commun. 2021 Oct 13;1(1):30–40. doi: 10.1158/2767-9764.CRC-21-0060 (PMC9973379; doi:10.1158/2767-9764.CRC-21-0060)
Supplement: Supplementary Table 1 — Target antigens, antibody clones, dilution of markers, and antigen retrieval conditions used for multiplex immunofluorescence staining [file crc-21-0060-s01.docx]

**Supplementary Table 1. Target antigens, antibody clones, dilution of markers, and antigen retrieval conditions used for multiplex immunofluorescence staining**

| Primary antibody | Clone ID/Company | Catalog # | Antibody  dilution | Fluor | Fluor dilution | Antigen retrieval, time (min) |
| --- | --- | --- | --- | --- | --- | --- |
| CD8 | 4B11 / Leica | NCL-L-CD8-4B11 | 1:200 | Opal 480 | 1:150 | ER1, 20 |
| PD-L1 | EL3N / Cell Signaling | 13684 | 1:300 | Opal 520 | 1:150 | ER1, 20 |
| FOXP3 | D608R / Cell Signaling | 12653 | 1:100 | Opal 570 | 1:300 | ER2, 40 |
| PD-1 | EPR4877(2) / Abcam | Ab137132 | 1:300 | Opal 620 | 1:300 | ER1, 20 |
| Cytokeratin | AE1/AE3 / Dako | M351529-2 | 1:100 | Opal 690 | 1:100 | ER1, 20 |
