# Supplementary material for: Comprehensive Immunoprofiling of High-Risk Oral Proliferative and Localized Leukoplakia
Source: Cancer Res Commun. 2021 Oct 13;1(1):30–40. doi: 10.1158/2767-9764.CRC-21-0060 (PMC9973379; doi:10.1158/2767-9764.CRC-21-0060)
Supplement: Supplementary Table 2 — Clinicopathologic predictors of cancer-free survival among patients with oral leukoplakia [file crc-21-0060-s02.docx]

**Supplementary Table 2.** **Clinicopathologic predictors of cancer-free survival among patients with oral leukoplakia**

| **Parameter** | **HR (95% CI), *N* = 58** | ***P*-value** |
| --- | --- | --- |
| Female vs. male gender | 0.54 (0.22-1.33) | 0.18 |
| Age at diagnosis | 1.00 (0.97-1.04) | 0.68 |
| History of smoking vs. not | 0.63 (0.42-1.13) | 0.75 |
| LL vs. PL  Primary oral site of disease^a^ | 11.25 (2.60-48.72)  0.73 (0.34-1.41) | **<0.01**  0.72 |
| Degree of dysplasia^b^  Time to first cancer diagnosis^c^ | 0.98 (0.56-1.72)  0.55 (0.31-1.09) | 0.95  0.84 |

^a^ compares oral tongue vs. palate, buccal, or alveolar gingiva vs. multifocal; ^b^ characterized as KUS = keratosis of undetermined significance vs. mild vs. moderate or severe dysplasia; ^c^ in months. HR = hazard ratio, CI = confidence interval, LL = localized leukoplakia, PL = proliferative leukoplakia. Multiple regression (Cox proportional hazard modeling) was only performed if *N* ≥ 10 patients were available in each subgroup. Testing of proportional-hazard assumptions yielded: Gonen and Heller’s K=0.97, Somers’ D=2K–1=0.95.
