# Supplementary material for: Comprehensive Immunoprofiling of High-Risk Oral Proliferative and Localized Leukoplakia
Source: Cancer Res Commun. 2021 Oct 13;1(1):30–40. doi: 10.1158/2767-9764.CRC-21-0060 (PMC9973379; doi:10.1158/2767-9764.CRC-21-0060)
Supplement: Supplementary Table 3 — Immunologic and molecular predictors of progression to cancer among oral leukoplakias [file crc-21-0060-s03.docx]

**Supplementary Table 3.** **Immunologic and molecular predictors of progression to cancer among oral leukoplakias**

| **Parameter** | **OR (95% CI), *N* = 58** | ***p*-value** |
| --- | --- | --- |
| CD8+ T cells  Macrophages  Natural killer cells  T regulatory cells | 1.20 (1.06-3.74)  1.93 (0.86-4.35)  1.96 (0.96-4.02)  2.30 (1.24-4.27) | 0.08  0.11  0.06  <0.01 |
| GZMM  CYLD  TCF7  CARD11  CCR7  ICOS  KLRB1  CD28  TRAF3  IKBKE | 2.99 (1.22-7.33)  9.01 (2.04-41.74)  6.29 (1.67-23.75)  2.42 (1.24-4.72)  2.16 (1.29-3.64)  1.65 (1.08-2.51)  2.67 (1.33-5.37)  3.11 (1.33-6.70)  1.63 (1.03-2.94)  3.98 (1.19-13.30) | 0.01  0.01  <0.01  <0.01  <0.01  0.02  <0.01  <0.01  0.03  0.02 |

Note: n=19 developed oral cavity SCC (2 with localized leukoplakia, LL and 17 with proliferative leukoplakia, PL). OR = odds ratio, CI = confidence interval. Binary multiple logistic regression analysis was only performed if n≥10 patients were available in each subgroup (for the purposes of analysis higher log2 expression values are correlated with progression to cancer)
